# Cytotoxic activity of genistein-8-C-glucoside form *Lupinus luteus* L. and genistein against human SK-OV-3 ovarian carcinoma cell line

**DOI:** 10.1007/s00044-016-1725-5

**Published:** 2016-10-03

**Authors:** Agata Antosiak, Katarzyna Milowska, Katarzyna Maczynska, Sylwia Rozalska, Teresa Gabryelak

**Affiliations:** 1 Department of General Biophysics, Faculty of Biology and Environmental Protection, University of Lodz, 141/143 Pomorska St., Lodz, 90-236 Poland; 2 Department of Industrial Microbiology and Biotechnology, University of Lodz, 12/16 Banacha St., Lodz, 90-237 Poland

**Keywords:** Genistein, Genistein-8-C-glucoside, Ovarian cancer, Cytotoxicity, Apoptosis

## Abstract

Genistein belongs to isoflavones, which are a subclass of flavonoids, a large group of polyphenolic compounds widely distributed in plants. Numerous in vitro studies suggest that isoflavones, particularly genistein, have both chemopreventive and chemotherapeutic potential in multiple tumor types. However, the molecular and cellular mechanisms of genistein effects on human ovarian cancer cells are still little known. In the present study, we investigated anticancer activity of genistein and its natural glucoside, genistein-8-C-glucoside isolated from flowers of *Lupinus luteus* L. We examined the effects of the two isoflavones alone or in combination on cultured human SK-OV-3 ovarian carcinoma cells. The cells were exposed to genistein and genistein-8-C-glucoside at various concentrations (1–90 µM) for 24 and 48 h. The cytotoxic and apoptotic properties of compounds were studied by the colorimetric 3-[4,5-2-yl]-2-5-diphenyltetrazolium bromide assay and the acridine orange/ethidium bromide staining technique. The morphological features of SK-OV-3 cells were examined by Nomarski differential interference contrast combined with a confocal laser scanning microscope. The level of ROS was evaluated with fluorescence probes: dichlorofluorescein-diacetate by flow cytometry. Changes in mitochondrial membrane potential were determined using 5,5,6,6-tetrachloro-1,1,3,3-tetraethylbenzimidazolcarbocyanine iodide. Genistein-treatment and genistein-8-C-glucoside-treatment resulted in the inhibition of cell proliferation, induction of apoptotic cell death and loss of mitochondrial membrane potential. The present data provide the first evidence in vitro that genistein-8-C-glucoside and combination genistein-genistein-8-C-glucoside could be a potential chemotherapeutic candidate for ovarian cancer therapy.

## Introduction

Epithelial ovarian cancer is the leading cause of death among gynecological malignancies in developed nations. With current protocols, although 70–80 % of patients respond to front-line chemotherapy, the majority relapses and ultimately succumbs to metastatic disease. The successful treatment of ovarian cancer depends greatly upon the effectiveness of cytotoxic anticancer drugs. Chemoresistance is related to relapse to ovarian cancer, but the underlying mechanisms are still poorly understood. Therefore, identification of novel agents against chemoresistant disease is still a major medical problem (Ahn et al. 2004; Hajra and Liu 2004; Gossner et al. 2007).

The flavonoids comprise a class of polyphenolic plant products that possess many biological properties offering possible new strategies for cancer chemotherapy (Lamartiniere 2000; Cooke et al. 2006; Klein and King 2007). Genistein (4′,5,7-trihydroxyisoflavone), a natural isoflavone phytoestrogen present in soybeans, is a potent agent in the prophylaxis and treatment of cancer. Like most isoflavones, genistein usually exists in nature in its 7-glycoside or 8-glycoside form, rather than in its aglycone form. Whether the isoflavones are biologically active in their glycosidic forms or require hydrolysis to their aglycone forms for activity is still a subject of some controversy (Choi et al. 2007).

Since the discovery by Akiyama et al. (1987) that genistein isolated from the fermentation broth of *Pseudomonas* species, inhibited the tyrosine-specific protein kinases of the epidermal growth factor (EGF) receptor, several in vitro studies have documented that genistein can inhibit the growth of various cancer cell lines: leukemia, lymphoma, prostate, breast, lung, and head or neck (Yoon et al. 2000; Sarkar and Li 2002; Klein and King 2007). It is generally believed that the free genistein aglycone undergoes absorption and exerts numerous biological activities, of which anticancer effects are abundantly proved (Polkowski et al. 2004). These may be mediated through the following mechanisms of action: induction of apoptosis, protein-tyrosine kinase induction, G2/M phase cell cycle arrest, inhibition of DNA topoisomerase II, suppression of telomerase activity, and inhibition of angiogenesis (Akiyama et al. 1987; Markovits et al. 1989; Buchler et al. 2003; Polkowski et al. 2004; Baxa et al. 2005). These activities, along with low toxicity, make genistein an important candidate for experimental therapy, as well as a new lead-compound for anticancer drug design. Although genistein’s molecular mechanisms of action have been studied in multiple tumor types (cell types), little has been elucidated with regard to its antineoplastic (anticancer) potential in ovarian cancer.

**Fig. 1:**
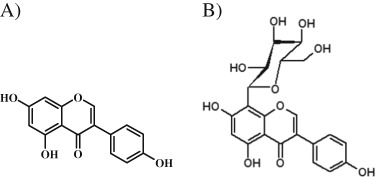
Chemical structure of genistein (**a**) and G8CG (**b**)

Studies have been made on various applications of genistein showing its high anticancerogenic potential. In spite of numerous therapeutic properties of genistein there are still many limitation, concerning its clinical application. They involve relatively low bioavailability of this compound, which a connection with two-phase action (in human cancer cell lines, genistein acts as a growth stimulator at low concentrations, and as a growth inhibitor at high concentrations) is a main reason why the practical use of genistein in cancer therapy is limited. Therefore, it is important to search for new derivatives of genistein (including other sources than soy) that can possess better physiochemical characterization or exhibit higher anticancerogenic activity. In this study, we investigated and compared the possible cytotoxic effects of genistein and its natural glycosylated derivative, genistein-8-C-glucoside (G8CG; Fig. 1) from *Lupinus luteus* L. in human SK-OV-3 ovarian carcinoma cells. G8CG-induced cytotoxicity in cancer cell lines has not yet been reported, but accounts of our previous studies (Rucinska et al. 2007; Rucinska and Gabryelak 2009) have suggested that G8CG has a biological activity. Therefore, we sought to determine whether these isoflavones alone or/and in combination exert a cytotoxic activity in ovarian cancer cells.

## Materials and methods

### Cell culture

Ovarian carcinoma cell line (SK-OV-3) was purchased from Child Health Centre in Warsaw (Poland). Cells were grown as a monolayer in McCoy’s 5a medium supplemented with 10 % fetal bovine serum with 100 units/ml gentamycin. The cultures were incubated at 37 °C in an atmosphere of 5 % CO_2_ and 95 % air with more than 95 % humidity. For experiments, exponentially growing cells were used. Cells growing as a monolayer culture were harvested by trypsinisation (0.25 % trypsin + 0.02 % EDTA). In our preliminary experiments such culture conditions were found to be optimal for maintaining the best parameters of cell proliferation and viability.

### Chemicals

Genistein, phosphate-buffered saline (PBS), dimethyl sulfoxide (DMSO), dichlorofluoroscein-diacetate (H_2_DCFDA), acridine orange (AO), ethidium bromide (EB), 3-[4,5-2-yl]-2-5-diphenyltetrazolium bromide (MTT), 5,5,6,6-tetrachloro-1,1,3,3-tetraethylbenzimidazolcarbocyanine iodide (JC-1), carbonylcyanide *p*-chloromethoxyphenylhydrazone (CCCP), were purchased from Sigma (St. Louis, MO, USA).

McCoy’s 5a medium, RPMI 1640 medium, trypsin, fetal bovine serum, and gentamycin were obtained from Gibco (BRL). Tissue culture dishes and flasks were purchased from Nunc (Roskilde, Denmark). All other reagents and solvents were of analytical grade.

### Chemical treatment of cells

G8CG was isolated from flowers of lupine (*Lupinus luteus* L.) according to the method developed by Laman and Volynets (1974). The isoflavon was subsequently extracted by methanol, ethylacetate, n-butanol and purified using a chromatographic column (3 × 80 cm) packed with polyamine and the column was washed subsequently with 15, 30, 50, and 80 % ethanol. G8CG was eluted by 50 % ethanol. G8CG was a beige powder with a purity of 97.5 % and was stored in a refrigerator (below −4 °C); protected from light, under nitrogen. Genistein and G8CG were taken from stock solution (500 μM) in 1 % DMSO. The concentration of DMSO used in this study as a vehicle had no effect on SK-OV-3 cell viability in our preliminary studies. Genistein or G8CG was added to the suspension of cultured cells to give a final concentration in the range of 1–90 μM. The cells were incubated with the isoflavones alone or in combination for 24 or 48 h at 37 °C. The control cells were treated with PBS (pH 7.4). All the fluorophores were dissolved in DMSO, and stored at −20 °C. JC-1 (1 μM final concentration) was added directly to the cell culture medium and incubated at 37 °C for the appropriate time, depending on the fluorophore.

### Cell proliferation assay

Proliferation of the cells was measured using the method of Mossman (1983). The cells were placed in 96-well microtiter plates at an initial density of 3 × 10^4^ cells in 200 μl per well. They were treated with genistein and G8CG (1–90 μM) at 37 °C in a 5 % carbon dioxide—95 % air atmosphere for 24 and 48 h, and recovered by gentle washing with PBS (pH 7.4) twice. After incubation, cell proliferation was determined by the MTT assay. MTT was dissolved in PBS at 5 mg/ml. Briefly, 50 μl of MTT solution was added to each well, followed by 4 h of incubation. After the incubation, MTT-containing medium was removed, and 100 μl of DMSO was added to each well to dissolve formazan crystals. Absorbance of the converted dye was measured at 545 nm with a correction at 630 nm using an enzyme-linked immunosorbent assay plate reader (Awarness Technology Inc.) Stat Fax Type. Cell viability was calculated as the percent ratio of absorbance of the samples to the referent control.

### Assessment of apoptosis

The percentage of apoptotic and viable cells was determined by the AO/EB staining technique described by Duke and Cohen (1992). An aliquot of 100 μl cell suspension (1 × 10^6^) previously exposed to genistein and G8CG at final concentrations in the range of 2.5–90 μM was added to the mixture of AO and EB (AO, 0.13 mM; EB, 0.23 mM) and incubated for 5 min at 37 °C in the dark. Fluorescent microscopy (Olympus IX70, Japan) was used to identify non-viable cells whose nuclei stained bright orange. Viable cells excluded EB and stained bright green. Quantitative assessments were made by determining the percentage of apoptotic cells, whose nuclei are highly condensed or fragmented. Three hundred images were randomly selected from each sample. Morphological evaluation of different stages of apoptosis was also analyzed in Nomarski DIC microscopy combined with confocal laser scanning microscope Pascal (Zeiss) equipped with Axiovert 200 upright microscope. Membrane blebs and condensed or pyknotic nuclei in the cells were regarded as indicators of apoptosis.

### Detection of mitochondrial membrane potential (Δψ_m_)

JC-1 is a lipophilic carbocyanine that exists in a monomeric form and is able to accumulate into mitochondria. In the presence of a high mitochondrial membrane potential (Δψ_m_), JC-1 can reversibly form aggregates which, after excitation at 488 nm, can be detected in the red channel (FL2). On the other hand, cells with low Δψ_m_ are those in which JC-1 maintains a monomeric form, showing green fluorescence (FL1). The ratio of red-green JC-1 fluorescence is dependent only on the mitochondrial membrane potential (Cossarizza et al. 1993). Cells were stained with the Δψ_m_-sensitive probe JC-1 used at the final concentration of 1 μM in fresh RPMI 1640 medium for 20 min at room temperature in the dark. The samples were washed twice by centrifuging at 500 g for 5 min with a double volume of PBS, and re-suspended in 0.5 ml of PBS, then immediately analyzed with the flow cytometer. JC-1 was excited at 488 nm, two different signals were collected on FACScan at 525 nm (FL1) and the aggregate signal (red) was analyzed at 590 nm (FL2).

### Determination of ROS generation

The level of reactive oxygen species (ROS) was stimulated with fluorescence probes-H_2_DCFDA by flow cytometry. H_2_DCFDA is a vital fluorescent probe that enters the cell and is hydrolyzed to dichlorofluorescein (DCF), whose interaction with peroxides gives rise to 2′,7′-dichlorofluorescin (Vanden Hoek et al. 1998). H_2_DCFDA was dissolved in DMSO at a concentration of 20 μM and stored at 4 °C. The cell suspension (1 × 10^6^) was treated with genistein and genistein-8-C-glycoside (from 2.5 to 90 μM) alone and in combination for 24 h at 37 °C. The cells were then washed twice in PBS (pH 7.4) and then gently re-suspended in RPMI 1640 medium and incubated for 30 min in the presence of 20 μM H_2_DCFDA. The fluorescence intensity of DCF was detected using flow cytometry (Becton Dickinson, LSR). The excitation and emission wavelengths were set at 488 and 530 nm, respectively.

### Statistical analysis

Data are presented as means±SD from at least five sets of measurements. The statistical difference between the control and treated groups was evaluated by the Student’s *t*-test. *p* < 0.05 and below was accepted as statistically significant.

## Results

### Antiproliferative activity of genistein and G8CG

**Fig. 2:**
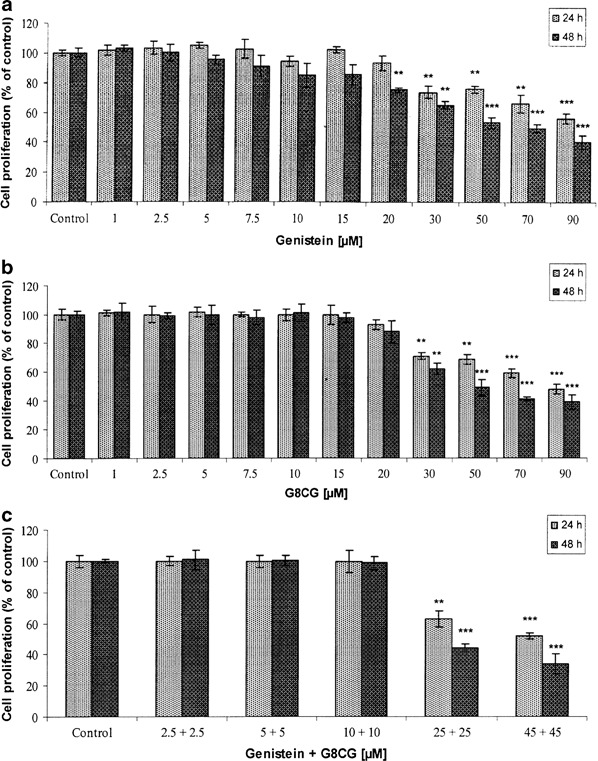
Cell proliferation after 24 or 48 h exposure of SK-OV-3 cells to: **a** genistein; **b** G8CG; **c** genistein-G8CG. The data are expressed as a percentage of the control value (value obtained for untreated cells). Each result represents mean±SD, *n* = 4, *p < 0.05, **p < 0.01, ***p < 0.001

The effects of genistein and G8CG either alone or in combination on the proliferation of SK-OV-3 cells were assessed by the MTT method. Genistein and G8CG significantly decreased cell proliferation after 24 or 48 h of exposure in a dose-dependent and time-dependent manner (*p* < 0.05, *p* < 0.01, *p* < 0.001, Figs. 2a, b). Treatment of SK-OV-3 cells with genistein and G8CG (1–20 µM) for 24 h did not affect cell proliferation, but the growing concentrations (>20 µM) decreased cell viability. Treatment with 90 µM G8CG inhibited cell proliferation by up to 52 % (24 h) and 61.1 % (48 h) compared to the control. When cells were treated with a combination of genistein and G8CG at 50 or 90 µM each, proliferative cell growth was blocked considerably (*p* < 0.01, *p* < 0.001, Fig. 2c). A 66.3 % (48 h) reduction in cell viability was observed for the 90 µM combination, which is similar to the decrease observed with 90 µM G8CG alone (61.1 %). Our results show that the tested compound demonstrates cytotoxic properties on estrogen-dependent SK-OV-3 cells.

### Apoptotic effect of genistein and G8CG

**Fig. 3:**
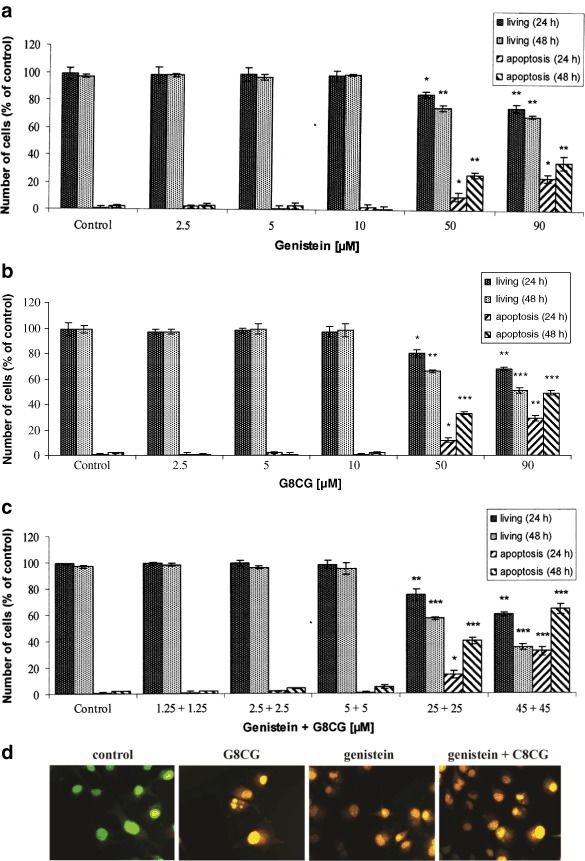
Induction of apoptosis after 24 or 48 h exposure of SK-OV-3 cells to: **a** genistein; **b** G8CG; **c** genistein-G8CG. The number of cells in individual experiments was 300. Each result represents mean±SD, *n* = 4, *p < 0.05, **p < 0.01, ***p < 0.001. Cells were treated with genistein, G8CG and genistein-G8CG combination (50 µM) for 24 h. The apoptosis was detected by AO/EB reaction technique, 400X; fluorescent microscopy and Nomarski DIC, CLSM, scale bar 20 µM (**d**)

The percentage of apoptotic cells detected by the AO/EB staining technique after treatment with genistein and G8CG either alone or in combination (2.5–90 µM) for 24 and 48 h was evaluated. Lower, physiological concentrations of genistein and G8CG (2.5–10 µM, 24–48 h) did not induce apoptotic cell death. The analysis indicated that the exposure to high concentrations of genistein and G8CG (50 or 90 µM) significantly affected apoptosis in a dose-dependent and time-dependent manner (*p* < 0.05, *p* < 0.01, *p* < 0.001, Figs. 3a, b). However, the apoptotic effect of genistein is weaker than that of G8CG at the same concentrations. Treatment with 90 µM G8CG increased the number of apoptotic cells by approximately 29 % (24 h) and 49 % (48 h) compared to untreated cells. A more pronounced, dose-dependent effect was observed after 48 h exposure to 50 or 90 µM genistein-G8CG combination; 40 and 64 % of apoptotic cells, respectively (*p* < 0.001, Fig. 3c). Apoptosis was also visualized by fluorescent microscopy (AO/EB assay) and confocal laser scanning microscopy (Contrast of Nomarski). The treatment of cells with genistein, G8CG, and genistein-G8CG combination (50 µM, 24 h) caused nuclei to become highly condensed or fragmented. Morphological features typical of apoptosis: gigantic cells with cytoplasmic bridges, nuclear condensation or fragmentation, membrane blebbing, and cytoplasmic granularity were also observed (Fig. 3d).

### Genistein and G8CG induce mitochondrial membrane depolarization

**Fig. 4:**
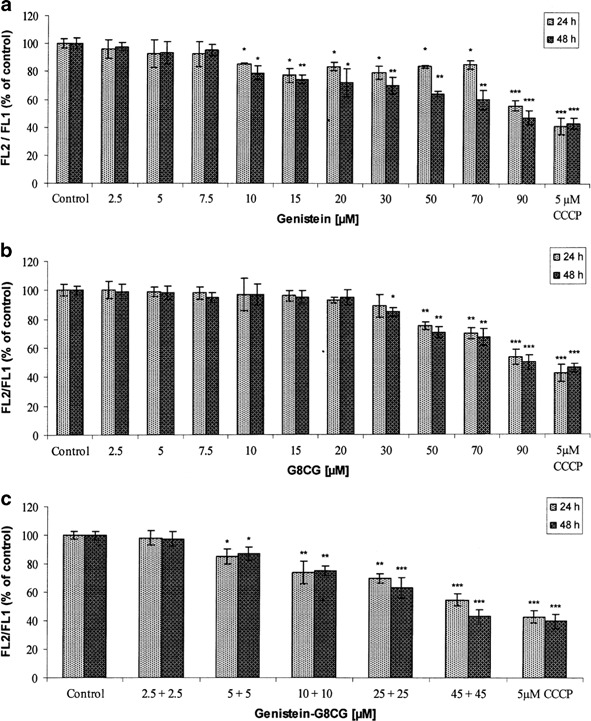
Loss of mitochondrial membrane potential (ΔΨ_m_) after 24 and 48 h exposure of SK-OV-3 cells to: **a** genistein; **b** G8CG; **c** genistein-G8CG. The calculated ratio of FL2 vs. FL1 of each sample is depicted. The data are expressed as a percentage of the control value (value obtained for untreated cells). Each result represents mean±SD, *n* = 4, *p < 0.05, **p < 0.01, ***p < 0.001

Changes in mitochondrial membrane potential (ΔΨ_m_) were determined by staining cells with the mitochondria-specific probe, JC-1. Genistein and G8CG significantly reduced ΔΨ_m_ after 24 or 48 h of exposure in a dose-dependent and time-dependent manner (*p* < 0.05, *p* < 0.01, *p* < 0.001, Figs. 4a, b). Treatment of SK-OV-3 cells with lower, physiological concentrations of genistein and G8CG for 24 and 48 h did not cause changes in the mitochondrial membrane potential. However, the exposure to high concentrations of genistein (≥10 µM) and G8CG (≥30 µM) strongly induced depolarization of ΔΨ_m_. Genistein and G8CG (90 µM) reduced mitochondrial membrane potential by 52.7 and 49 % (48 h), respectively. A more dramatic drop in the red fluorescence was observed after 48 h exposure to 50 or 90 µM genistein-G8CG combination; 36.8 and 56.8 %, respectively (*p* < 0.01, *p* < 0.001, Fig. 4c). These findings suggest that mitochondria could be a primary target for genistein-induced and G8CG-induced apoptosis and that loss of ΔΨ_m_ is an early event in the process.

#### ROS generated by genistein and G8CG

**Fig. 5:**
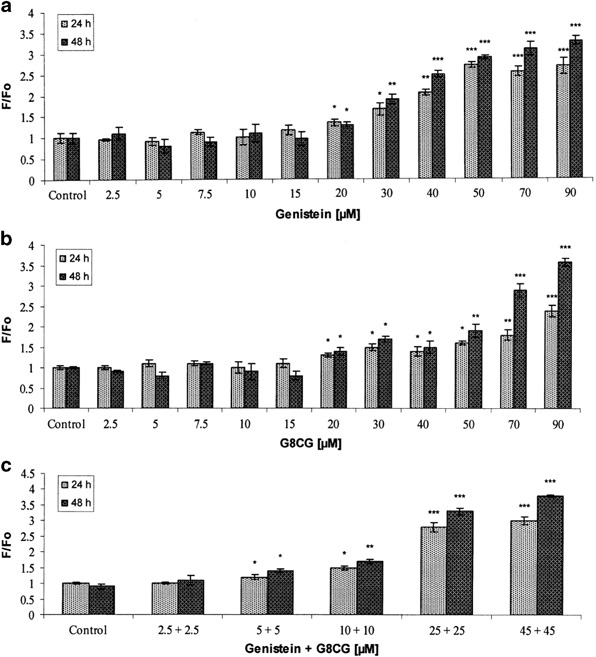
ROS production after 24 and 48 h exposure of SK-OV-3 cells to: **a** genistein; **b** G8CG; **c** genistein-G8CG. ROS production was measured as DCF fluorescence. Each result represents mean±SD, *n* = 4, *p < 0.05, **p < 0.01, ***p < 0.001

Generation of ROS was verified by the measurement of changes in fluorescent intensity of H_2_DCFDA resulting from intracellular probe oxidation. Fluorescence intensity can be easily measured and it is the basis of the popular cellular assay for oxidative stress. The effects of genistein and G8CG either alone or in combination on H_2_DCFDA fluorescence is shown in Figs. 5a–c, respectively (*p* < 0.05, *p* < 0.01, *p* < 0.001). The results show that high concentrations of genistein or G8CG (≥20 µM) and genistein-G8CG combination (≥10 µM) increased DCF fluorescence, which suggests that these isoflavones can cause DCF oxidation by generating reactive species, in particular H_2_O_2_. In contrast, after the treatment with lower concentrations of the compounds (2.5–20 µM), the rate of ROS generation was not different from the rate observed in normal cells. The obtained results show that the treatment of SK-OV-3 cells with genistein and G8CG generates ROS, including H_2_O_2_, suggesting that these flavonoids can induce ROS-dependent apoptosis.

## Discussion

Epithelial ovarian cancer is a very common malignancy in industrialized nations and is highly chemoresistant to currently available chemotherapeutic agents. In a large screening, in vitro studies have documented that numerous isoflavonoids could prevail over the resistant capacity and display effective antiproliferative activities in various cancer cell lines. Dietary relevant sources of isoflavonoids, represented by genistein, are soybeans and soy derived foodstuffs. The interest in genistein originated from epidemiological findings indicating that it may provide protection against chronic diseases such as hormone-dependent cancers (Lee et al. 1991; Adlercreutz et al. 1993) and disorders of cardiovascular system (Setchell and Cassidy 1999). Subsequent studies revealed that genistein exhibits multiple pharmacological effects, so at present the isoflavone is emerging as an active ingredient for nutraceuticals or as a prospective drug candidate for anticancer therapy (Polkowski and Mazurek 2000; Dixon and Ferreira 2002; Popiołkiewicz et al. 2005). The estrogenic activity of genistein and its subsequent modulation of cellular processes in tumorigenic ovarian cells are not well characterized.

In the present study, we were the first to investigate anticancer activity of free aglycone genistein and its natural glucoside, G8CG isolated from flowers of *Lupinus luteus* L. in cultured human SK-OV-3 ovarian carcinoma cells. We examined the effect of the two isoflavones alone or in combination in various concentrations (2.5–90 µM, 24 or 48 h) on cell proliferation, induction of apoptotic cell death, mitochondrial membrane potential and the level of ROS in cells. It has been reported that low-dose concentrations of genistein, comparable to physiological levels achieved with dietary intake of isoflavones including genistein, produce a stimulatory effect on estrogen-dependent human breast cancer cells in vitro and in vivo (Allred 2001; Ju 2001). To determine whether this effect was also seen in ovarian cancer cells, SK-OV-3 cells were treated with physiological concentrations of genistein and G8CG (1–10 µM) for 24–48 h. In our studies, low-dose genistein and G8CG treatment did not result in proliferation of ovarian cancer cells when compared to the control. We demonstrated that high doses of genistein significantly decreased cell proliferation, which was consistent with previously published reports indicating that genistein may be an effective agent against cancer. Genistein has been reported to inhibit the proliferation of several cancer cell lines. Our observations are in agreement with in vitro results in several ovarian tumor cells: A2780, CaOV3, ES2, and SK-OV-3 (Choi et al. 2007; Gossner et al. 2007), which indicated that high, supraphysiological concentrations (>10 µM) of genistein exhibit a cytotoxic activity. Like genistein, G8CG and a combination of genistein-G8CG at high concentrations inhibited proliferation of SK-OV-3 cells, suggesting that G8CG possesses an antiproliferative effect similar to that of non-glycosylated genistein. Recently, our research provided direct evidence that G8CG from *Lupinus luteus* L. is able to inhibit proliferation of hamster ovary CHO cells and mouse NIH 3T3 cells in vitro (Rucinska et al. 2007, 2008).

Inhibition of cell proliferation has been associated with induction of apoptosis in other cell types (Allred 2001). In epithelial cells, apoptosis is an early response to cell death. Although genistein has been shown to induce apoptosis in a variety of human cancer cells, its mechanism of action is not fully known. Some recent works provide evidence that genistein can induce not only apoptosis, but also autophagic cell death, in overlapping or parallel scenarios. Gossner et al. (2007) observed that the mechanism of genistein-induced cell death involves both apoptosis and autophagy. Because autophagy is typically an adaptive response to nutrient starvation, scientists hypothesized that genistein could induce a starvation-like signaling response. We have now demonstrated that the treatment of SK-OV-3 cells with genistein and G8CG (50 or 90 µM, 24 or 48 h) induced apoptotic cell death in a dose-dependent and time-dependent manner, which was consistent with the literature. We also postulate that genistein and G8CG combined are more effective than genistein alone. It is possible that apoptosis induction by G8CG may have a different pathway than genistein. This apoptotic effect may be explained by difficult concentrations; i.e., high doses of isoflavones may produce a deleterious effect (Leopold et al. 1976; Setchell et al. 1987) and induce apoptosis through mitochondrial-dependent pathways. Yoon et al. (2000) demonstrated that genistein (90 µM, 24 h) induced cytochrome *c* release, caspase-3 activation, nuclear condensation and DNA fragmentation by reducing the mitochondrial membrane potential. Lower, but not quite physiological concentrations of genistein (15–60 µM) induced apoptosis in murine T-cell lymphoma cell lines via mitochondrial depolarization (Baxa et al. 2005). In recent years, numerous studies have demonstrated that most, if not all, cells experience a collapse of their mitochondrial membranes as a prelude to nuclear DNA degradation and apoptosis (Ravagnan et al. 2002). Indeed, mitochondria are now thought to act as key coordinators of apoptosis (Green and Reed 1998). In agreement with this, we have shown here that, in SK-OV-3 cells, genistein and G8CG alone or in combination induce depolarization of the mitochondrial membrane potential. Lower concentrations of isoflavones (<10 µM) did not induce mitochondrial depolarization. It is questionable, whether genistein would have any effect on mitochondrial-dependent apoptosis at physiologically relevant levels (Klein and King 2007). Our data let us suggest that mitochondria could be one of the targets for the action of genistein and its derivative.

Increasing evidence suggests that ROS can also induce apoptotic cell death and can act as a target for anticancer strategy. In this study, the data showed that genistein and G8CG (≥20 µM, 24 and 48 h) induced a significant increase in ROS production and loss of ΔΨ_m._ Because ROS are well recognized to act as secondary messengers in diverse intracellular signaling cascades, they may be the main molecules responsible for the observed cytotoxic effects (Yeh et al. 2007). Cao et al. (1997) proposed a reaction sequence for ROS production by flavonoids, whose first step requires the oxidized state of involved transition metal, which is decreased by reacting with the flavonoids in a reduced form. Mitochondrial DNA is particularly susceptible to damage by ROS because of its close proximity to the electron transport chain and its lack of protective histones. Prolonged dissipation of ΔΨ_m_ might be attributed to this susceptibility to mitochondrial DNA damage. Taken together, the data suggest that an interaction with mitochondria might partially explain the apoptotic cell death during a long-term exposure to genistein or G8CG.

In summary, our studies demonstrated that genistein and/or G8CG exerted multiple suppressive effects on SK-OV-3 cells, including proliferation inhibition, induction of apoptosis, collapse of mitochondrial membrane potential, and generation of ROS. Most importantly, our data provide the first evidence that G8CG and genistein-G8CG combination could be potential candidates for ovarian cancer therapy. These findings provided a basis for further investigation on genistein and G8CG for the treatment and prevention of ovarian cancer.

## Abbreviations

G8CG: genistein-8-C-glucoside
DIC: differential interference contrast
SK-OV-3: human ovarian carcinoma cells
EGF: epidermal growth factor
PBS: phosphate-buffered saline
DMSO: dimethyl sulfoxide
H_2_DCFDA: dichlorofluoroscein-diacetate
AO: acridine orange
EB: ethidium bromide
MTT: 3-[4,5-2-yl]-2-5-diphenyltetrazolium bromide
JC-1: 5,5,6,6-tetrachloro-1,1,3,3-tetraethylbenzimidazolcarbocyanine iodide
CCCP: carbonylcyanide *p*-chloromethoxyphenylhydrazone
